# A Case of Gonorrhœa, with Some Nuts for the Isopaths to Crack

**Published:** 1883-01

**Authors:** E. W. Berridge

**Affiliations:** London


### CLINICAL BUREAU.

### A CASE OF GONORRHŒA, WITH SOME NUTS FOR THE ISOPATHS TO CRACK.

E. W. BERRIDGE, M. D., M. I. H. A.

Before commenting on a system, it is necessary to define it. What is isopathy? Some, seeking the explanation of the word in a Greek lexicon, define it as the method of treating a disease by the administration of the *same* virus that produced it; hence they point to the allopathic method of syphilization as a case in point, because here the crude virus is used to cure the effects of the crude virus; while, on the other hand, they declare that to give dynamized *Syphilinum* is not isopathy, because the potency is different from the crude material. But this argument proves too much. By parity of reasoning, Homœopathy must signify merely healing by a remedy producing symptoms similar to those of the patient, irrespective of all rules concerning dynamization, etc. No true homœopathician will admit this. To fully comprehend the meaning of either of these words, we must go to the founder of each system and learn what he meant by it.

HAHNEMANN founded a system and called it Homœopathy; Homœopathy, therefore, logically signifies HAHNEMANN'S system; and this definition remains true, whether the system be proved erroneous or not. Similarly organopathy logically means the system so named by Loeffler and afterward rehashed by Sharp. So again isopathy must mean the system founded by Lux, who gave it this name. Now *Lux* did not recommend the *crude*, but the *dynamized* virus; hence to treat diseases even by this dynamized product may be not Homœopathy but isopathy; at any rate, even if we consider the allopathic processes of syphilization and variolation as isopathic, Lux's method is not thereby removed from that category by reason of its employment of *dynamized* nosodes.

What, then, is the radical difference between isopathy and Homœopathy?

The homœopath treats the individual patient.

The isopath treats the disease.

The homœopath individualizes.

The isopath generalizes.

The homœopath prescribes for the totality of the symptoms.

The isopath prescribes for one, the chief objective, symptom.

The homœopath values the subjective symptoms.

The isopath necessarily ignores them.

The homœopath says: "The totality of the symptoms of this case corresponds to the symptoms of *Syphilinum;* therefore, whether the case be syphilitic or not, *Syphilinum* is the *simillimum.*"

The isopath says: "This case is syphilitic, therefore, *Syphilinum* must be given, whether the totality of the symptoms indicates it or not."

Which of these two systems is *a priori* the most scientific it is unnecessary to state.

In my former paper on "The Scientific Use of the Nosodes," I quoted a series of cases to show how unsatisfactory in practice isopathy must be. Out of ten nosodes with which I had tested the matter, the most striking results were obtained from *Psoriasinum* in psoriasis. I explained this case, because some seemed to disposed to run to a contrary extreme, and, in opposition to those who maintained that the nosode would cure every case of the corresponding disease, asserted that it would never cure any, which is equally an error. But since then I have seen *Psoriasinum* tried in another obstinate case of psoriasis *without the slightest effect.* What more evidence is required to prove that isopathy is a mockery, a delusion, and a snare. But to my case of gonorrhœa.

In November, 1881, a gentleman consulted me for a second attack of gonorrhœa. I could elicit no *very* characteristic symptoms. Suffice it to say that I gave him high potencies of *Mercurius, Medorrhinum, Canth., Argent nitr., Nux, Kali bichr., Thuja, Cannab.,* with *much* relief. The medicines were all carefully selected from the provings, and they relieved temporarily, but did not cut the disease short, as the simillimum would have done. At last he gave me the key-note to the case; he had painful erections *when riding in the railway cars,* except when he was engaged in conversation. This symptom belongs to *Calc. phosph.,* of which I gave him seven doses of CM (Fincke) one daily for seven days. The remedy acted at once, removed this distressing symptom and also the remains of the gonorrhœa. I would ask the isopaths why *Medorrhinum* did not cure this case. According to their doctrine it ought to have done so. The answer is that *Medorrhinum* did not correspond to the totality of the symptoms and *Calc. phosph.* did. This necessary correspondence can only be obtained by accurate and extensive provings, and without these our success with nosodes, though it may at times be startling, yet is empirical, and may at any time fail us. It has been said that every case of disease is a proving of the nosode. Granted that it contains the symptoms of the nosode, but are they pure? In some cases they are, in some they are not; and, like all other provings, they need clinical verification or pathogenitic confirmation before they can be unreservedly accepted.

Now I ask the champions of isopathy this question: Clinical experience shows that a nosode will *cure* some cases of the disease, *relieve in part* others, and *utterly fail* in others. *How can we tell, therefore, in what cases to give it?* Theoretically I hold that the cases it will cure are uncomplicated cases. But how are we to diagnose them with unerring accuracy? It is impossible; and what we need is careful provings with the dynamized preparations, which I am convinced will be found among the most powerful of our weapons. When we have these we can compare them with the symptoms attributed to the crude virus.

This has been already done to some extent. In Hering's monograph of *Lyssinum* we can compare the cured symptoms with those of the potencies, and from the like. In *Guiding Symptoms* we see the symptoms of anthrax compared with those cured by *Anthracinum*, though provings with the potencies are greatly needed. Cures by *Syphilinum* have been published by Dr. Swan in the *Homœopathic World*, and may be compared with his provings, which are now in type for that journal. I have a large collection of provings of *Medorrhinum*, with many cured cases, and only delay copying them for press till I receive the *complete* report from one of the best of the provers. Will not some of the members of I. H. A. solve this burning question by proving the nosodes?

In the meantime, as our enemies accuse us of forsaking Homœopathy for isopathy, I propose the following resolution, to be voted on at the next meeting of our Association:

"*Resolved*, That we repudiate Lux's system of isopathy as a departure from Homœopathy, and an empirical and hazardous method of prescribing according to the name of the disease instead of according to the symptoms of the individual patient; and that we do not indorse any use of the nosodes except the prescribing of them according to accurately observed pathogenesis supplemented where necessary by carefully verified clinical experience."

