# Errata

**Published:** 1883-03

**Authors:** 


Errata.—In our January number, page 36, line 17 from top, for *explained* read *emphasized;* page 37, line 21 from top, for *like* read *bite.* This latter correction is of importance.

